# Marker-Based Pose Estimation of End Effectors in Industrial Robot Visual Servoing: Error Modeling and Placement Guidelines

**DOI:** 10.3390/s26134124

**Published:** 2026-06-30

**Authors:** Xuewen Wei, Pengcheng Li, Pinzhang Wang, Yunfei Miao, Wei Tian, Wenhe Liao

**Affiliations:** 1 College of Mechanical & Electrical Engineering, Nanjing University of Aeronautics and Astronautics, Nanjing 210016, China; 2 School of Mechanical Engineering, Nanjing University of Science and Technology, Nanjing 210094, China

**Keywords:** visual servoing, marker-based pose estimation, pose estimation accuracy, error propagation, rigid registration, marker placement

## Abstract

In industrial robot visual servoing, the accuracy of end-effector pose directly affects the feedback quality and trajectory tracking performance of the visual servo system. To improve end-effector pose estimation accuracy, this paper establishes a propagation model from marker measurement errors to end-effector pose estimation errors and further derives the covariance expression of pose estimation errors. Based on this model, different marker placement factors affecting translational and rotational errors are analyzed, including marker-set spatial range, spatial distribution balance, and centroid offset distance. In addition, the influence of the number of markers on pose estimation errors is derived by adding a new marker to an existing point set. The accuracy of the analytical model is validated through Monte Carlo simulations and experiments, and guidelines for marker placement and marker number are provided.

## 1. Introduction

Industrial robot visual servo machining can fully exploit the flexibility of industrial robots and compensate for the adverse effects caused by their limited absolute positioning accuracy through closed-loop feedback control [1]. In such systems, the target-state information provided by visual sensors serves as a critical feedback signal for closed-loop control, and its accuracy directly affects trajectory-tracking performance and task execution quality. When errors exist in the target pose, the feedback information received by the control system deviates from the true state. This increases trajectory tracking errors, reduces machining accuracy, and may even affect system stability and robustness. Therefore, improving the accuracy of target pose under visual feedback is essential for enhancing the performance of industrial robotic machining based on visual servoing.

In visual servoing systems, visual feedback is typically composed of image features extracted from the target or artificial fiducial markers. According to their representation, commonly used visual features can be classified into local and global features. Local features include points, lines, corners, circle centers, and marker centers, which generally correspond to local geometric elements of the target [2]. Global features include image moments, area, centroid, and principal-axis orientation, which are commonly used to characterize the overall shape and pose of the target region [3]. In industrial robotic applications requiring three-dimensional target pose estimation, artificial fiducial markers are widely used in visual measurement and visual servoing because of their reliable detectability, explicit geometric constraints, and suitability for establishing rigid-body relationships. Typical artificial fiducial markers include AprilTag, ArUco, circular, and retroreflective markers [4]. This study focuses on end-effector pose estimation using multiple markers. Specifically, the visual system acquires the coordinates of multiple markers rigidly attached to the end-effector, and the end-effector pose is subsequently determined through rigid registration.

Improving the measurement accuracy of individual markers is a direct way to enhance target pose estimation accuracy. Following this idea, extensive studies have been conducted mainly from three aspects: marker design, image processing and feature extraction, and stereo vision calibration. First, markers have been encoded to improve their identifiability and the stability of subpixel extraction, thereby reducing the effects of illumination variations, occlusion, and noise [5]. Second, target detection networks, attention mechanisms, and center extraction procedures have been optimized to improve marker detection and localization accuracy in complex backgrounds [6]. Third, more accurate distortion models have been established, and stereo calibration configurations have been optimized to improve system calibration and three-dimensional reconstruction accuracy [7,8]. These studies have significantly improved the measurement accuracy of individual markers.

In addition to analyses of the measurement error of individual markers, the propagation of marker measurement errors through rigid point registration to uncertainty in the registration result has also been extensively investigated in medical navigation and optical tracking. Fitzpatrick et al. [9] established the classical analytical relationship between fiducial localization error and target registration error (TRE), and revealed the effects of the number of markers, point-set scale, moments of inertia, and the position of the target point relative to the point-set centroid on TRE. Subsequent studies extended this framework to biased, heterogeneous, anisotropic, and weighted marker errors, leading to more general first-order frameworks for predicting rigid registration errors [10]. Other studies incorporated factors more closely related to practical applications by combining marker localization error with target localization error, tool calibration error, and reference-frame error. Consequently, TRE models have gradually evolved from early theoretical formulations into error-assessment tools that are more applicable to engineering and clinical scenarios [11,12,13,14]. In recent years, Zhe et al. [15,16] further advanced this line of research by proposing the concept of total target registration error (TTRE). Pivot calibration uncertainty and target localization error (TLE) in both spaces were explicitly incorporated into the analytical framework. These studies established the theoretical foundation for predicting rigid point-registration errors, with primary emphasis on TRE, fiducial registration error (FRE), target-point localization error, and marker localization errors with different statistical characteristics.

Compared with studies that primarily use TRE as the evaluation metric, some studies have directly investigated the estimation errors of rigid-body translation and rotation. Davis et al. [17] analyzed the rotation matrix error and translation vector error induced by marker errors using first-order error propagation. Danilchenko et al. [18] further established a general propagation relationship from marker errors to six-dimensional rotational–translational errors. Franaszek et al. [19] investigated the covariance and directional characteristics of rotational uncertainty. In the fields of industrial robotics and visual measurement, Li et al. [20] analyzed the influence of the offset between the marker-set centroid and the origin of the end-effector coordinate frame on end-effector pose. Bertels et al. [21] used Monte Carlo simulations to investigate the effects of point-set volume, number, arrangement, and camera parameters on six-degree-of-freedom pose accuracy. Wu et al. [22] and Jiang et al. [23] improved the stability of visual localization from the perspectives of marker visibility, target structure, and dynamic tracking, respectively. Overall, research on target pose errors has gradually expanded from the error of an individual target point to the overall pose error of the target coordinate frame, with progress made in theoretical derivation, simulation analysis, and experimental evaluation. However, for industrial robot visual servoing, there is still a lack of systematic studies that analyze marker-set spatial range, distribution balance, centroid offset, and marker number within a unified six-dimensional pose-error covariance framework, while separately revealing their different effects on end-effector translational and rotational errors. In particular, regarding the influence of marker number on target pose errors, existing studies have mainly relied on TRE scaling relationships under specific geometric conditions or on simulation and experimental comparisons among point sets with different numbers of markers. A direct theoretical analysis of its influence on the complete six-dimensional target pose error is still lacking.

To address this issue, this study investigates the effects of marker placement and marker number on target pose estimation errors. First, starting from the rigid-registration-based target pose estimation process, the error propagation relationship between marker position measurement errors and target pose estimation errors is established, and the corresponding covariance expression is further derived. Based on the resulting error model, the effects of marker spatial range, spatial distribution balance, and centroid offset distance on translational and rotational estimation errors are systematically analyzed. To investigate the influence of marker number, a single-marker incremental analysis based on nested point sets is adopted to prove that, under the assumed marker-error conditions, adding a marker to an existing point set cannot increase either the translational or rotational estimation error. Finally, the theoretical analysis is validated through simulations and experiments, based on which guidelines for marker placement in industrial robot visual servoing are proposed.

The main contributions of this study are threefold. First, using the end-effector and camera coordinate frames commonly adopted in industrial robot visual servoing, this study reformulates the established first-order error propagation framework. The resulting model directly characterizes the translation and rotation errors in end-effector pose feedback and facilitates the application of the theoretical results to marker-placement design. Second, a unified, factor-oriented analytical framework is established to distinguish the different effects of marker-set spatial range, spatial distribution balance, centroid offset, and marker number on translation and rotation estimation errors. Third, regarding the influence of marker number, a single-marker incremental analysis based on nested point sets is adopted. Under the marker-error assumptions employed in this study, it is proved that adding a marker with the same measurement accuracy to an existing point set does not increase either the translation or rotation estimation error. This conclusion does not require the geometric statistics of the point set, such as the distances from the markers to the principal axes of the marker set, to remain unchanged.

The remainder of this paper is organized as follows. Section 2 presents the propagation relationship between marker measurement errors and end-effector pose estimation errors based on rigid-body registration, and derives the covariance expression of the pose estimation errors. Section 3 analyzes the effects of marker-set spatial range, spatial distribution balance, centroid offset distance, and the number of markers on end-effector pose estimation errors based on the established error model. Section 4 and Section 5 validate the accuracy of the proposed error model through Monte Carlo simulations and experiments, respectively, and illustrate the marker-placement guidelines derived for different marker configurations. Section 6 summarizes the paper and presents the main conclusions.

## 2. Error Propagation Model from Marker Points to the End-Effector Pose

### 2.1. System Description and Problem Definition

**Figure 1:**
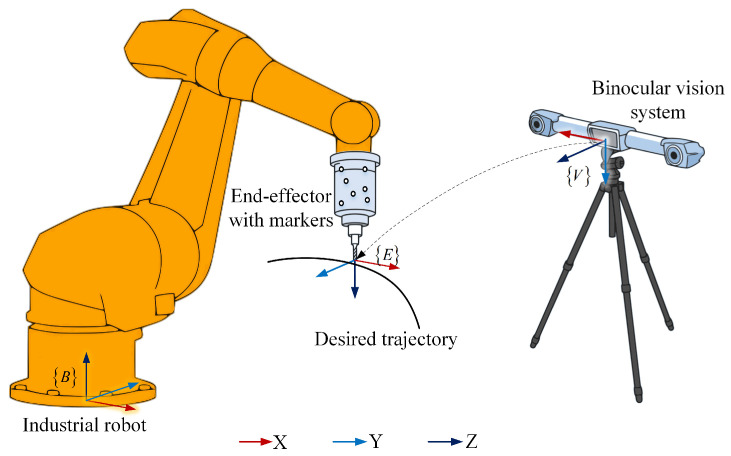
Schematic diagram of end-effector pose estimation in industrial robot visual servoing. The arrows labeled X, Y, and Z at the bottom serve as a legend for the color-coded positive directions of the coordinate axes.

Figure 1 shows the end-effector pose measurement relationship in industrial robot visual servoing. The system mainly consists of a six-degree-of-freedom industrial robot, an end effector, and a binocular vision system for real-time pose feedback. The industrial robot carries the end effector to track a predefined Cartesian trajectory, while the binocular vision system measures the real-time pose of the end effector. To describe the pose measurement process, three key coordinate systems are defined: the robot base coordinate system $\left\{B\right\}$, the end-effector coordinate system $\left\{E\right\}$, and the binocular camera coordinate system $\left\{V\right\}$. Several markers are fixed on the end effector. The binocular vision system measures the three-dimensional coordinates of these markers in real time, and the pose of the end effector relative to the binocular camera is then obtained through rigid registration. The measured pose is transformed into the robot base coordinate system and used as feedback information for the visual servo controller, enabling closed-loop correction of the end-effector motion state. Therefore, the accuracy of end-effector pose measurement directly affects the feedback quality and trajectory tracking performance of the visual servo system. This paper focuses on how to improve the pose measurement accuracy of the end effector through appropriate marker placement.

### 2.2. Error Propagation and Covariance Expression for End-Effector Pose Measurement

**Figure 2:**
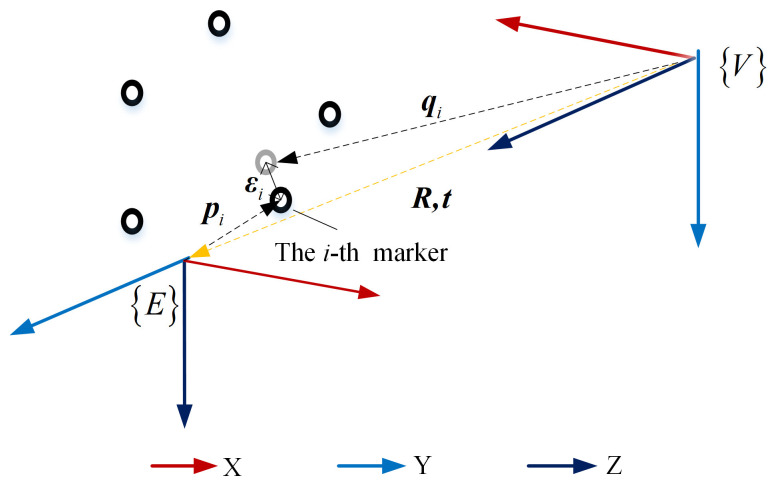
Schematic diagram of marker-based rigid registration. The arrows labeled X, Y, and Z at the bottom serve as a legend for the color-coded positive directions of the coordinate axes.

The first-order propagation relationship from marker errors to rigid-body translational and rotational errors has been established in studies on marker-based tracking, rigid point registration, and combined visual-tracking measurements [17,18]. In this study, this relationship is reformulated using notation suitable for the subsequent analysis of marker placement. Figure 2 illustrates marker-based rigid registration using a total of n markers fixed on the end effector. Let $R\in SO\left(3\right)$ and $t\in R^{3}$ denote the true rotation matrix and translation vector, respectively, from the end-effector coordinate system $\left\{E\right\}$ to the camera coordinate system $\left\{V\right\}$. For the i-th physical marker, $p_{i}$ denotes its known coordinate in $\left\{E\right\}$. In the absence of visual measurement error, the coordinate of this marker in $\left\{V\right\}$ would be $Rp_{i}+t$. Let $\varepsilon_{i}$ denote the visual measurement error of the i-th marker, defined as the deviation of its measured coordinate from the corresponding ideal coordinate. Denoting the measured coordinate of the same marker in $\left\{V\right\}$ by $q_{i}$, the following relationship is obtained

(1) $$q_{i}=Rp_{i}+t+\varepsilon_{i}$$

Let the translation and rotation estimates obtained by rigid registration be $\hat{t}$ and $\hat{R}$, respectively. Their relationships with the true values t and R can be expressed as:

(2) $$\hat{t}=t+\delta t$$

(3) $$\hat{R}=\left(I_{3}+{\left[\delta \theta \right]}_{\times }\right)R$$

where δt and δθ are the errors propagated from marker measurement errors to the end-effector pose through rigid registration. These errors are referred to as estimation errors in this study, with δt and δθ denoting the translational estimation error and rotational estimation error, respectively. δθ is a small rotation vector expressed in radians. ${\left[\delta \theta \right]}_{\times }$ is the cross-product matrix corresponding to δθ:

(4) $$\delta t=\left[\begin{array}{c} \delta t_{x}\\ \delta t_{y}\\ \delta t_{z} \end{array}\right],\;\delta \theta =\left[\begin{array}{c} \delta \theta_{x}\\ \delta \theta_{y}\\ \delta \theta_{z} \end{array}\right],\;{\left[\delta \theta \right]}_{\times }=\left[\begin{array}{ccc} 0 & -\delta \theta_{z} & \delta \theta_{y}\\ \delta \theta_{z} & 0 & -\delta \theta_{x}\\ -\delta \theta_{y} & \delta \theta_{x} & 0 \end{array}\right]$$

The estimated coordinate of the i-th marker in the camera coordinate system can be expressed as:

(5) $${\hat{q}}_{i}=\hat{R}p_{i}+\hat{t}$$

Substituting Equations (2) and (3) into Equation (5) gives:

(6) $${\hat{q}}_{i}=Rp_{i}+t-{\left[Rp_{i}\right]}_{\times }\delta \theta +\delta t$$

$$r_{i}=q_{i}-{\hat{q}}_{i}\cdot \delta e={\left[\begin{array}{cc} \delta t & \delta \theta \end{array}\right]}^{T}\cdot J_{i}=\left[\begin{array}{cc} I_{3} & -{\left[Rp_{i}\right]}_{\times } \end{array}\right]$$

The registration error is defined as $r_{i}=q_{i}-{\hat{q}}_{i}$, the end-effector pose estimation error is defined as $\delta e={\left[\begin{array}{cc} \delta t^{T} & \delta \theta^{T} \end{array}\right]}^{T}$, and $J_{i}=\left[\begin{array}{cc} I_{3} & -{\left[Rp_{i}\right]}_{\times } \end{array}\right]$ is introduced. The vector δe is introduced solely to provide a compact representation of the joint propagation of marker measurement errors to rigid-body translation and rotation estimation errors. It is not used to define a unified Euclidean norm or a scalar measure of the six-degree-of-freedom pose estimation error. Combining Equations (1) and (6) gives:

(7) $$r_{i}=\varepsilon_{i}-J_{i}\delta e$$

Define $\varepsilon ={\left[\begin{array}{cccc} \varepsilon_{1}^{T} & \varepsilon_{2}^{T} & \cdots & \varepsilon_{n}^{T} \end{array}\right]}^{T}$, $r={\left[\begin{array}{cccc} r_{1}^{T} & r_{2}^{T} & \cdots & r_{n}^{T} \end{array}\right]}^{T}$, and $J={\left[\begin{array}{cccc} J_{1}^{T} & J_{2}^{T} & \cdots & J_{n}^{T} \end{array}\right]}^{T}$. Following the form of Equation (7), the following expression can be obtained:

(8) $$r_{i}=\varepsilon_{i}-J_{i}\delta e$$

If the registration process is exact, the registration error satisfies r=0. Therefore, Equation (8) can be rewritten as:

(9) ε−Jδe=0

However, due to the presence of visual measurement errors, exact registration cannot be achieved. Equation (9) is an overdetermined equation, and its least-squares solution is:

(10) $$\delta e={\left(J^{T}J\right)}^{-1}J^{T}\varepsilon$$

Equation (10) represents the propagation relationship from marker measurement errors to end-effector pose estimation errors. Let the covariance matrix associated with ε be denoted by $\Sigma_{\varepsilon }$. The covariance matrix corresponding to the end-effector pose estimation error can then be obtained as:

(11) $$\Sigma_{\delta e}={\left(J^{T}J\right)}^{-1}J^{T}\Sigma_{\varepsilon }J{\left(J^{T}J\right)}^{-1}$$

Previous studies have considered more general cases involving biased, heterogeneous, anisotropic, and weighted marker errors [16,18]. The present study focuses on isolating and analyzing the influence of marker placement on end-effector pose estimation errors. To avoid confounding the effects of marker geometry with differences in the error magnitudes and directional characteristics of individual markers, a controlled and uniform measurement-error condition is adopted. Specifically, for each marker, the measurement errors in the x, y, and z directions are mutually independent and follow the normal distribution $N\left(0,\sigma^{2}\right)$. Under this assumption, Equation (11) can be rewritten as:

(12) $$\Sigma_{\delta e}={\left(J^{T}J\right)}^{-1}\sigma^{2}$$

If the marker set can sufficiently constrain the end-effector coordinate system, $J^{T}J$ is a real symmetric positive definite matrix. Solving Equation (12) gives:

(13) $$\Sigma_{\delta e}=\left[\begin{array}{cc} \frac{1}{n}I_{3}+R{\left[c\right]}_{\times }G_{p}^{-1}{\left[c\right]}_{\times }^{T}R^{T} & R{\left[c\right]}_{\times }G_{p}^{-1}R^{T}\\ -RG_{p}^{-1}{\left[c\right]}_{\times }R^{T} & RG_{p}^{-1}R^{T} \end{array}\right]\sigma^{2}$$

where $c=\frac{1}{n}\sum_{i=1}^{n}p_{i}$ is the centroid offset vector of the marker set relative to the origin of the end-effector coordinate system, hereafter referred to as the centroid offset vector. Its norm $\left\|c\right\|$ is referred to as the centroid offset distance. The matrix $G_{p}$ is given by:

(14) $$G_{p}=\sum_{i=1}^{n}\left({\left\|{\tilde{p}}_{i}\right\|}^{2}I_{3}-{\tilde{p}}_{i}{\tilde{p}}_{i}^{T}\right)$$

(15) $${\tilde{p}}_{i}=p_{i}-c$$

It should be noted that $G_{p}$ is fully determined by the marker coordinates $p_{i}$ in the end effector and the centroid offset vector c. Therefore, it is an intrinsic property of the marker set and is independent of the camera coordinate system. This matrix is equivalent to the moment-of-inertia matrix of the point set about its centroid. Similar geometric matrices have been used to analyze the relationships between point-set configuration and TRE and rotational uncertainty [9,19]. For convenience in the subsequent analysis, this matrix is referred to as the point-set information matrix. Since $J^{T}J$ is a real symmetric positive definite matrix, $G_{p}$ is also a real symmetric positive definite matrix.

## 3. Analysis of Marker Placement Factors Affecting End-Effector Pose Estimation Error

### 3.1. Effect of Marker Spatial Distribution on End-Effector Pose Estimation Error

(1) Effect of marker spatial distribution on rotational estimation error

According to Equation (13), the covariance of the rotational estimation error can be expressed as:

(16) $$\Sigma_{\theta \theta }=RG_{p}^{-1}R^{T}\sigma^{2}$$

The rotational error covariance expression is consistent with the corresponding results reported in previous first-order analyses of point-registration errors and rotational uncertainty [18,24]. The subsequent analysis focuses on using its eigenstructure to explain the effects of different marker-placement factors. The total variance of the rotational estimation error is defined as the evaluation metric for the rotational estimation error:

(17) $$E_{r}=tr\left(\Sigma_{\theta \theta }\right)$$

Let the three eigenvalues of $G_{p}$ be $\lambda_{1},\lambda_{2},\lambda_{3}$. Since $G_{p}$ is a real symmetric positive definite matrix, all its eigenvalues are positive. Without loss of generality, assume that

(18) $$0<\lambda_{\min}=\lambda_{1}\leq \lambda_{2}\leq \lambda_{3}=\lambda_{\max}$$

Substituting Equation (16) into Equation (17) gives:

(19) $$E_{r}=\left(\frac{1}{\lambda_{1}}+\frac{1}{\lambda_{2}}+\frac{1}{\lambda_{3}}\right)\sigma^{2}$$

Equation (19) shows that the rotational estimation error is determined by the eigenvalues of $G_{p}$. Since $G_{p}$ is constructed from the marker coordinates, the spatial distribution of the markers determines the eigenvalue distribution of $G_{p}$. The following discussion is conducted from two aspects: the marker-set spatial range and the spatial distribution balance.

(a) Effect of spatial range

First, under the condition that the spatial distribution balance of the marker set remains unchanged, the effect of spatial range variation on the rotational estimation error is analyzed. The marker coordinates are scaled as:

(20) $${\tilde{p}}_{i}^{\prime }=\zeta {\tilde{p}}_{i}$$

where ζ>1 is the scaling factor.

The information matrix corresponding to the marker set after coordinate scaling is:

(21) $$G_{p}^{\prime }=\sum_{i=1}^{n}\left({\left\|\zeta {\tilde{p}}_{i}\right\|}^{2}I_{3}-\zeta {\tilde{p}}_{i}{\tilde{p}}_{i}^{T}\zeta \right)=\zeta^{2}\sum_{i=1}^{n}\left({\left\|{\tilde{p}}_{i}\right\|}^{2}I_{3}-{\tilde{p}}_{i}{\tilde{p}}_{i}^{T}\right)=\zeta^{2}G_{p}$$

Furthermore, it can be obtained that

(22) $$E_{r}^{\prime }=tr\left(R{\left(G_{p}^{\prime }\right)}^{-1}R^{T}\sigma^{2}\right)=tr\left(R{\left(\zeta^{2}G_{p}\right)}^{-1}R^{T}\sigma^{2}\right)=\frac{1}{\zeta^{2}}E_{r}$$

Equation (22) shows that a larger marker-set spatial range leads to a smaller corresponding rotational estimation error.

(b) Effect of spatial distribution balance

Furthermore, under the condition that the marker-set spatial range remains unchanged, the effect of spatial distribution balance variation on the rotational estimation error is analyzed. The trace of $G_{p}$ can be expressed as:

(23) $$tr\left(G_{p}\right)=\sum_{i=1}^{n}\left(tr\left({\left\|{\tilde{p}}_{i}\right\|}^{2}I_{3}\right)-tr\left({\tilde{p}}_{i}{\tilde{p}}_{i}^{T}\right)\right)=\sum_{i=1}^{n}\left(3{\left\|{\tilde{p}}_{i}\right\|}^{2}-{\left\|{\tilde{p}}_{i}\right\|}^{2}\right)=2\sum_{i=1}^{n}{\left\|{\tilde{p}}_{i}\right\|}^{2}$$

On the other hand, according to the property of the matrix trace,

(24) $$tr\left(G_{p}\right)=\lambda_{1}+\lambda_{2}+\lambda_{3}$$

Combining Equations (23) and (24) gives:

(25) $$\lambda_{1}+\lambda_{2}+\lambda_{3}=2\sum_{i=1}^{n}{\left\|{\tilde{p}}_{i}\right\|}^{2}$$

The physical meaning of $\sum_{i=1}^{n}{\left\|{\tilde{p}}_{i}\right\|}^{2}$ is the sum of squared distances from the markers to the centroid of the marker set. It characterizes the spatial range of the marker set. In this paper, its square root is defined as the marker-set spatial range metric:

(26) $$S_{p}=\sqrt{\sum_{i=1}^{n}{\left\|{\tilde{p}}_{i}\right\|}^{2}}$$

Equation (25) shows that, when the spatial range of the markers is fixed, the sum of the eigenvalues of $G_{p}$ is fixed. In this case, the variation in $E_{r}$ essentially depends on how the three eigenvalues are distributed. According to Jensen’s inequality, $E_{r}$ reaches its minimum when $\lambda_{1}=\lambda_{2}=\lambda_{3}$. A more unbalanced distribution of the three eigenvalues leads to a larger $E_{r}$, and $E_{r}$ is particularly sensitive to smaller eigenvalues. The relationship between the eigenvalue distribution of $G_{p}$ and the spatial distribution balance of the marker set is then analyzed. The following quadratic form is defined:

(27) $$Q\left(v\right)=v^{T}G_{p}v$$

Substituting Equation (14) into Equation (27) gives:

(28) $$Q\left(v\right)=\sum_{i=1}^{n}\left({\left\|{\tilde{p}}_{i}\right\|}^{2}{\left\|v\right\|}^{2}-{\left({\tilde{p}}_{i}^{T}v\right)}^{2}\right)=\sum_{i=1}^{n}{\left\|{\tilde{p}}_{i}\times v\right\|}^{2}$$

where v is a unit vector, and $\left\|{\tilde{p}}_{i}\times v\right\|$ represents the perpendicular distance from point ${\tilde{p}}_{i}$ to vector v. Therefore, $Q\left(v\right)$ represents the sum of squared distances from all markers in the marker set to vector v. According to the Rayleigh quotient property, $\lambda_{\min}\leq Q\left(v\right)\leq \lambda_{\max}$. When v is the eigenvector corresponding to $\lambda_{\min}$, $Q\left(v\right)=\lambda_{\min}$. When v is the eigenvector corresponding to $\lambda_{\max}$, $Q\left(v\right)=\lambda_{\max}$.

Two cases of spatial distribution balance are considered. If the spatial distribution of the marker set is relatively balanced, the sums of squared distances from the marker set to different directions are also relatively close. Very small or very large values do not occur. This means that the eigenvalues of $G_{p}$ are relatively uniformly distributed. If the marker set is highly unbalanced, for example, many markers are close to a certain axis or the marker set exhibits an elongated shape, the sums of squared distances from the marker set to some directions are small. In contrast, those in the other directions are large. This means that the eigenvalue distribution of $G_{p}$ is highly uneven, with a very small minimum eigenvalue and a very large maximum eigenvalue. Therefore, the spatial distribution balance of the marker set is positively correlated with the uniformity of the eigenvalue distribution of $G_{p}$.

In summary, when the marker-set spatial range is fixed, the spatial distribution balance of the marker set affects the rotational error by influencing the uniformity of the eigenvalue distribution of $G_{p}$. A more balanced marker set leads to a more uniform eigenvalue distribution of $G_{p}$, and thus a smaller total variance of the rotational estimation error. Conversely, a more unbalanced marker set leads to a more uneven eigenvalue distribution of $G_{p}$, and thus a larger total variance of the rotational estimation error. In particular, the direction in which the marker set is most concentrated corresponds to the eigenvector direction associated with the minimum eigenvalue of $G_{p}$. Along this direction, the geometric support of the marker set is the weakest, and its influence on the rotational error is the greatest. This direction is referred to as the principal axis direction of the marker set in this paper.

(2) Effect of marker spatial distribution on translational estimation error

According to Equation (13), the covariance of the translational estimation error can be expressed as:

(29) $$\Sigma_{\mathrm{tt}}=\left(\frac{1}{n}I_{3}+R{\left[c\right]}_{\times }G_{p}^{-1}{\left[c\right]}_{\times }^{T}R^{T}\right)\sigma^{2}$$

Classical TRE studies have shown that the position of the target point relative to the centroid of the marker set affects the target-point position error [9,18]. Studies on robotic vision measurement have also demonstrated that, when the coordinate-frame origin deviates from the marker-set centroid, rotational perturbations are coupled into the translational measurement result [20]. This study further quantitatively analyzes this coupling term within the covariance framework of the translational parameter error. The total variance of the translational estimation error is defined as the evaluation metric for the translational estimation error:

(30) $$E_{t}=tr\left(\Sigma_{\mathrm{tt}}\right)$$

Substituting Equation (29) into Equation (30) gives:

(31) $$E_{t}=\left(\frac{3}{n}+\mathrm{tr}\left({\left[c\right]}_{\times }^{T}G_{p}^{-1}{\left[c\right]}_{\times }\right)\right)\sigma^{2}$$

Let $u_{i},\;i=1,2,3$, be the unit eigenvectors of $G_{p}$. Equation (31) can also be written in the following form:

(32) $$E_{t}=\left(\frac{3}{n}+\sum_{i=1}^{3}\frac{{\left\|c\times u_{i}\right\|}^{2}}{\lambda_{i}}\right)\sigma^{2}$$

Equation (32) shows that the translational error depends not only on the eigenvalues of $G_{p}$, but also on the relative relationship between the centroid offset vector c and the eigenvectors of $G_{p}$. The following discussion is conducted from three aspects: the marker-set spatial range, spatial distribution balance, and centroid offset distance.

(a) Effect of spatial range

First, under the condition that the spatial distribution balance and centroid offset of the marker set remain unchanged, the effect of spatial range variation on the translational estimation error is analyzed. Following the analysis of the rotational estimation error, after the marker coordinates are scaled by ζ, the marker-set information matrix becomes $G_{p}^{\prime }=\zeta^{2}G_{p}$. Accordingly, its eigenvalues become $\zeta^{2}$ times the original eigenvalues, while the eigenvectors remain unchanged. According to Equation (32), $E_{t}$ decreases.

(b) Effect of spatial distribution balance

Furthermore, under the condition that the marker-set spatial range and centroid offset remain unchanged, the effect of spatial distribution balance variation on the translational estimation error is analyzed. From Equation (32), $\left\|c\times u_{i}\right\|$ physically represents the perpendicular distance from the centroid of the marker set to the eigenvector direction $u_{i}$ of the information matrix $G_{p}$. Furthermore, $\sum_{i=1}^{3}{\left\|c\times u_{i}\right\|}^{2}/\lambda_{i}$ can be interpreted as the weighted sum of squared distances from the centroid of the marker set to the three eigenvector directions of $G_{p}$, with weights of $1/\lambda_{i}$. When the marker-set spatial range is fixed, changes in the spatial distribution balance of the marker set alter the eigenvalue distribution and eigenvector directions of $G_{p}$. If the marker set is relatively balanced, the three eigenvalues of $G_{p}$ are close to each other, and the corresponding weights $1/\lambda_{i}$ are also close. In this case, the translational estimation error is less sensitive to the direction of the centroid offset vector.

In contrast, if the marker set is unbalanced, the eigenvalue distribution of $G_{p}$ also becomes unbalanced. According to the analysis of the rotational estimation error, the principal axis direction of the marker set corresponds to the eigenvector direction associated with the minimum eigenvalue. Therefore, the corresponding weight $1/\lambda_{i}$ in this direction is the largest. If the centroid offset direction is approximately perpendicular to the principal axis direction of the marker set, the distance from the centroid to this direction is large. The corresponding weight is also large, which significantly increases the total variance of the translational estimation error. If the centroid offset direction is approximately parallel to the principal axis direction of the marker set, the distance from the centroid to this direction is small. In this case, even if the corresponding weight is large, its influence on the translational estimation error is weak. The effect of marker spatial distribution balance on the translational estimation error does not have general monotonicity. It depends on the relative relationship between the centroid offset vector and the characteristic directions of the marker set. The closer the principal axis direction of the marker set is to the centroid offset direction, the more favorable it is for reducing the translational error.

(c) Effect of centroid offset distance

Finally, under the condition that the spatial distribution balance and spatial range of the marker set remain unchanged, the effect of centroid offset distance variation on the translational estimation error is analyzed. The centroid offset distance is scaled as:

(33) $$c^{\prime }=\zeta c$$

where ζ>1 is the scaling factor.

Let the total variance of the translational estimation error corresponding to the new centroid offset vector be $E_{t}^{\prime }$. According to Equation (32), the variation in the total variance of the translational estimation error can be obtained as:

(34) $$\Delta E_{t}=E_{t}^{\prime }-E_{t}=\left(\zeta^{2}-1\right)\sum_{i=1}^{3}\frac{{\left\|c\times u_{i}\right\|}^{2}}{\lambda_{i}}$$

Since ζ>1 and $\lambda_{i}>0$, $\Delta E_{t}>0$. This indicates that, when the spatial distribution balance and spatial range of the marker set remain unchanged, a larger centroid offset distance leads to a larger translational estimation error.

### 3.2. Effect of the Number of Markers on End-Effector Pose Estimation Error

Previous studies have mainly analyzed error-scaling behavior under specific geometric conditions through the marker-number term in TRE formulations, or examined the influence of marker number using simulations and experiments with different numbers of markers [9,21]. Rather than directly comparing two point sets that differ in both size and configuration, this section considers nested point sets. Specifically, a new marker is added while retaining all markers in the original point set, and the effect of the additional observation on translational and rotational estimation errors is analyzed based on the complete six-dimensional pose information matrix.

Consider an original point set containing n markers. A new marker $p_{n+1}$ is added while retaining all the original markers, resulting in a new point set containing n+1 markers. According to the definition of the Jacobian matrix for an individual marker in Section 2, the Jacobian matrix corresponding to the newly added marker $p_{n+1}$ is:

(35) $$J_{n+1}=\left[\begin{array}{cc} I_{3} & -{\left[Rp_{n+1}\right]}_{\times } \end{array}\right]$$

For any vector $l\in R^{6}$, $l^{T}J_{n+1}^{T}J_{n+1}l={\left\|J_{n+1}l\right\|}_{2}^{2}\geq 0$. Therefore, $J_{n+1}^{T}J_{n+1}$ is a real symmetric positive semidefinite matrix.

In the following, the superscripts $\left(n\right)$ and $\left(n+1\right)$ are used to denote the quantities associated with the original and new point sets, respectively. For example, the Jacobian matrices of the two point sets are denoted by $J^{\left(n\right)}$ and $J^{\left(n+1\right)}$, respectively. According to Equation (12), the covariance matrices of the end-effector pose estimation error corresponding to the two point sets before and after the addition of a new marker are given by, respectively:

(36) $$\Sigma_{\delta e}^{\left(n\right)}={\left({\left(J^{\left(n\right)}\right)}^{T}J^{\left(n\right)}\right)}^{-1}\sigma^{2}$$

(37) $$\Sigma_{\delta e}^{\left(n+1\right)}={\left({\left(J^{\left(n+1\right)}\right)}^{T}J^{\left(n+1\right)}\right)}^{-1}\sigma^{2}$$

Since the new point set retains all the markers in the original point set, its Jacobian matrix can be expressed as $J^{\left(n+1\right)}={\left[\begin{array}{cc} {\left(J^{\left(n\right)}\right)}^{T} & J_{n+1}^{T} \end{array}\right]}^{T}$, it then follows that:

(38) $${\left(J^{\left(n+1\right)}\right)}^{T}J^{\left(n+1\right)}={\left(J^{\left(n\right)}\right)}^{T}J^{\left(n\right)}+J_{n+1}^{T}J_{n+1}\;$$

According to Section 2.2, under the condition that the original marker set sufficiently constrains the end-effector pose, ${\left(J^{\left(n\right)}\right)}^{T}J^{\left(n\right)}$ is a real symmetric positive-definite matrix. Since $J_{n+1}^{T}J_{n+1}$ is a real symmetric positive-semidefinite matrix, it follows from Equation (38) that ${\left(J^{\left(n+1\right)}\right)}^{T}J^{\left(n+1\right)}\;$ is also a real symmetric positive-definite matrix, and its relationship with ${\left(J^{\left(n\right)}\right)}^{T}J^{\left(n\right)}$ can be expressed as ${\left(J^{\left(n+1\right)}\right)}^{T}J^{\left(n+1\right)}\;\underline{≻}\;{\left(J^{\left(n\right)}\right)}^{T}J^{\left(n\right)}$. Furthermore, their inverse matrices satisfy ${\left({\left(J^{\left(n+1\right)}\right)}^{T}J^{\left(n+1\right)}\right)}^{-1}\;\underline{≺}\;{\left({\left(J^{\left(n\right)}\right)}^{T}J^{\left(n\right)}\right)}^{-1}$. Here, “$\underline{≻}$” and “$\underline{≺}$” denote the positive-semidefinite ordering between matrices. For two real symmetric matrices A and B of the same dimension, $A\;\underline{≻}\;B$ means that A−B is positive semidefinite, whereas $A\;\underline{≺}\;B$ means that A−B is negative semidefinite. Combining Equation (36) and Equation (37) gives:

(39) $$\Sigma_{\delta e}^{\left(n+1\right)}\;\underline{≺}\;\Sigma_{\delta e}^{\left(n\right)}$$

The covariance matrices of the translational and rotational estimation errors are the corresponding principal submatrices of $\Sigma_{\delta e}$, and therefore preserve the same positive-semidefinite ordering. According to the definitions of $E_{t}$ and $E_{r}$ in Section 3.1, taking the traces of the corresponding covariance matrices gives:

(40) $$E_{t}^{\left(n+1\right)}\leq E_{t}^{\left(n\right)}$$

(41) $$E_{r}^{\left(n+1\right)}\leq E_{r}^{\left(n\right)}$$

Therefore, adding a new marker to the original point set does not increase the translational or rotational estimation error of the end-effector; these errors can only decrease or remain unchanged.

## 4. Simulation Validation of Marker Placement Effects on End-Effector Pose Estimation Error

### 4.1. Simulation Framework

A simulation framework was established in a numerical computing environment. First, the marker coordinates in the end-effector coordinate system and the true pose of the end-effector coordinate system relative to the camera coordinate system were given. According to the rigid transformation relationship, the true coordinates of each marker in the camera coordinate system were calculated. Random measurement noise was then added to obtain the measured marker coordinates. Subsequently, the estimated pose of the end-effector coordinate system relative to the camera coordinate system was obtained from the measured marker coordinates using the classical SVD-based least-squares rigid registration method [25]. The detailed algorithm is provided in Appendix A. By comparing the estimated pose with the true pose, the simulation results of the end-effector pose estimation error were obtained. For each marker set, the above process was repeated for M Monte Carlo simulations. The simulation parameters were set as follows: the measurement noise variance of a single marker in each coordinate direction was $\sigma^{2}=0.01\;{\mathrm{mm}}^{2}$; the true translation of the end-effector coordinate system relative to the camera coordinate system was $t={\left[100,50,2000\right]}^{T}\mathrm{mm}$; the true orientation was represented by ZYX Euler angles as ${\left[10,30,-10\right]}^{T}\deg$, which were converted into the rotation matrix R in the simulation. The number of Monte Carlo simulations was set to M=5000.

### 4.2. Accuracy Validation of the Error Model for End-Effector Pose Measurement

**Figure 3:**
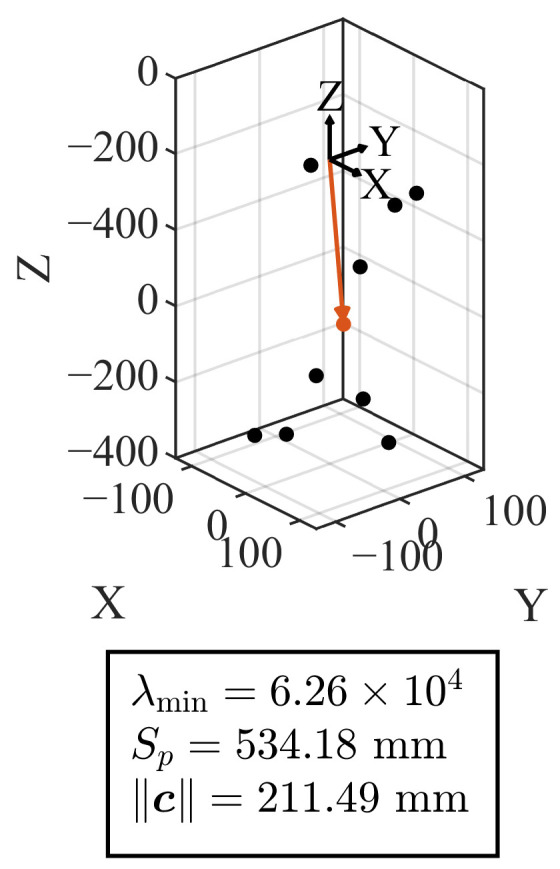
Spatial distribution of markers for model accuracy validation.

To validate the effectiveness of the end-effector pose estimation error model under general conditions, a non-symmetric marker set with a nonzero centroid offset distance was selected as the simulation object. The spatial distribution of this marker set in the end-effector coordinate system is shown in Figure 3. The black points denote the markers, the red point denotes the marker-set centroid, and the red vector denotes the centroid offset vector. In addition, several properties of the marker set are annotated below the figure, including the minimum eigenvalue $\lambda_{\min}$ of the marker-set information matrix, which is used to quantify the spatial distribution balance of the marker set, the marker-set spatial range $S_{p}$, and the centroid offset distance $\left\|c\right\|$. Subsequently, based on the simulation framework described in Section 4.1, samples of the end-effector pose estimation error were obtained, and their sample covariance matrix was calculated:

(42) $$\Sigma_{\delta e}^{MC}=\frac{1}{M-1}\sum_{k=1}^{M}\left(\delta e_{k}-\bar{\delta e}\right){\left(\delta e_{k}-\bar{\delta e}\right)}^{T}$$

where $\bar{\delta e}$ is the sample mean of the end-effector pose estimation error, and the diagonal elements of $\Sigma_{\delta e}^{MC}$ are the sample variances of the error components. On the other hand, the analytical result of the end-effector pose estimation error covariance matrix can be calculated according to Equation (13), denoted as $\Sigma_{\delta e}^{A}$.

Table 1 compares the Monte Carlo simulation results with the analytical results. The first six items correspond to the variances of the six components of δe, and their relative differences are calculated as the absolute difference between the simulation value and the analytical value divided by the analytical value. The last item is the relative Frobenius norm error, which is used to evaluate the overall difference between the covariance matrix obtained from Monte Carlo simulations and the analytical covariance matrix. It is calculated as $\left({\left\|\Sigma_{\delta e}^{MC}-\Sigma_{\delta e}^{A}\right\|}_{F}/{\left\|\Sigma_{\delta e}^{A}\right\|}_{F}\right)\times 100\%$. The simulation results show that the relative differences in the variances of all error components are less than 3%, and the relative Frobenius norm error of the pose estimation error covariance matrix is also less than 3%. Therefore, the Monte Carlo simulation results are in good agreement with the analytical results, which verifies the accuracy of the analytical model established in Section 2 for predicting end-effector pose estimation errors.

### 4.3. Simulation Analysis of Marker Spatial Distribution Effects

(1) Marker spatial distribution balance

**Figure 4:**
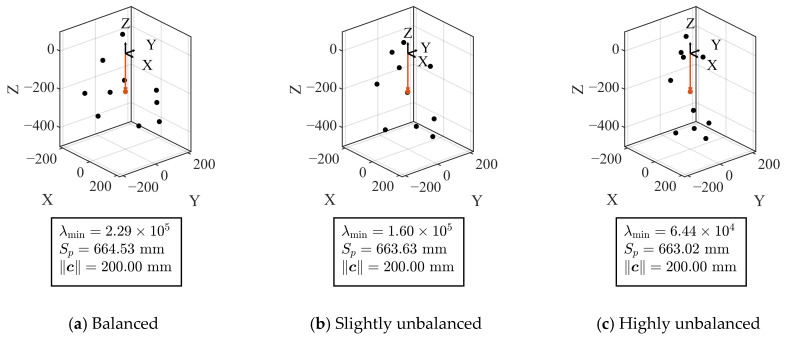
Marker sets with different spatial distribution balances.

**Figure 5:**
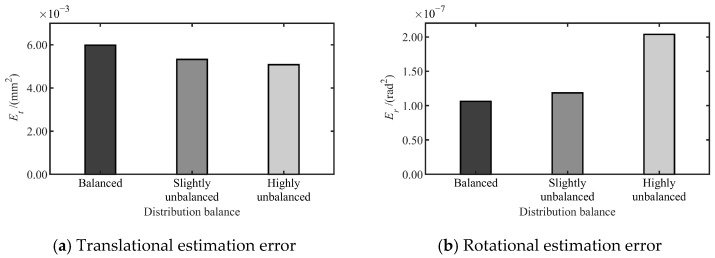
Pose estimation error metrics for different distribution balances under the original centroid offset condition.

To analyze the effect of marker spatial distribution balance on end-effector pose estimation error, three marker sets were selected, as shown in Figure 4. The marker sets progressively converge toward the z-axis in space, representing a gradual transition from a balanced distribution to an unbalanced distribution. Monte Carlo simulations were conducted for the three marker sets, and the translational and rotational estimation error metrics were calculated. The results are shown in Figure 5.

**Figure 6:**
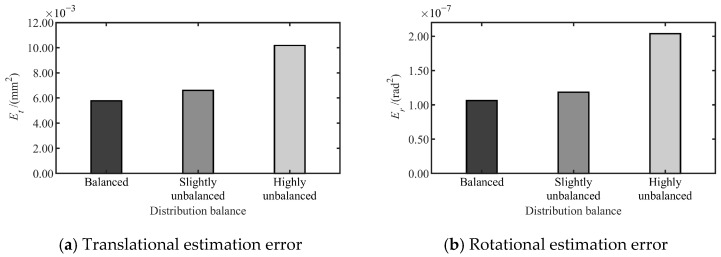
Pose estimation error metrics for different distribution balances after changing the centroid offset direction.

The simulation results show that, as the marker set gradually converges toward the z-axis of the target coordinate system, the rotational error increases, whereas the translational error decreases. According to the analysis in Section 3.1, convergence of the marker set toward the z-axis decreases the eigenvalue of $G_{p}$ in the z-axis direction and increases the corresponding weight in this direction. However, since the centroid offset vector of the marker set is approximately parallel to the z-axis direction, the distance from the centroid to the z direction is close to zero. Therefore, the increased weight in the z-axis direction has a weak effect on the translational estimation error. On the other hand, when the marker-set spatial range remains unchanged, the sum of the eigenvalues remains constant. A decrease in the eigenvalue in the z direction means that the eigenvalues in the other characteristic directions increase, thereby reducing the corresponding weights in these directions. As a result, the translational estimation error decreases. To further verify this conclusion, the marker set is translated as a whole while keeping the marker-set spatial range and balance unchanged, so that the centroid offset vector becomes (−200, 0, 0). In this case, the centroid offset direction is approximately perpendicular to the principal axis direction of the marker set. Theoretically, translating the marker set as a whole does not change the relative distribution of the markers. Therefore, the rotational errors of the three marker sets should remain consistent with those before translation. However, because the centroid offset direction is changed, the translational error increases as the marker set converges toward the z-axis. Figure 6 shows the simulation results after modifying the centroid offset vector, which are consistent with the above theoretical analysis.

(2) Marker-set spatial range

**Figure 7:**
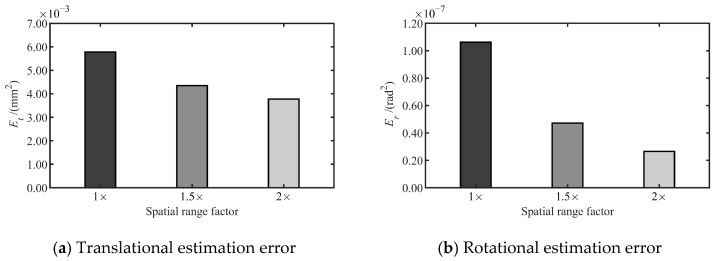
Pose estimation error metrics for marker sets with different spatial ranges.

The marker set in Figure 4a was used as the baseline marker set. The distances from each marker to the marker-set centroid were scaled by factors of 1.5 and 2 to generate two new marker sets. The statistical results of the translational and rotational estimation errors corresponding to different marker sets are shown in Figure 7. The results show that both translational and rotational estimation errors gradually decrease as the marker-set spatial range increases, and the decrease in rotational estimation error is more significant.

(3) Centroid offset distance

**Figure 8:**
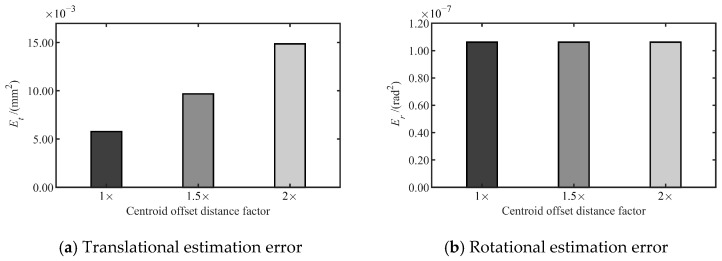
Pose estimation error metrics for different centroid offset distances.

The marker set in Figure 4a was used as the baseline marker set. The marker set was translated as a whole so that the centroid offset distance was scaled by factors of 1.5 and 2, generating two new marker sets. The statistical results of the translational and rotational estimation errors corresponding to different marker sets are shown in Figure 8. The results show that, as the centroid offset distance of the marker set increases, the translational estimation error increases significantly, whereas the rotational estimation error remains almost unchanged.

### 4.4. Simulation Analysis of Marker Number Effects

To analyze the effect of the number of markers on pose estimation error, seven nested marker sets were constructed. The first set contains the first four markers. Each subsequent set was generated by adding two markers to the previous set, until the seventh set contained 16 markers. The coordinates of all markers are listed in Table 2. The newly added markers were arranged in an approximately centrally symmetric manner to avoid significant changes in the spatial distribution balance of the marker set as the number of markers increases. Meanwhile, the centroids of all marker sets were controlled near (0, 0, −200). In addition, considering the structural size of the end effector, all markers were located within a spherical region centered at the marker-set centroid with a radius of 200 mm.

**Figure 9:**
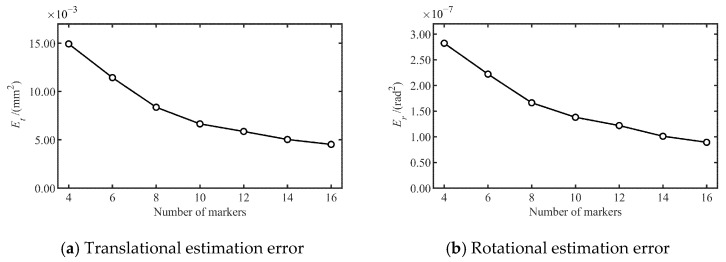
Simulated pose estimation error metrics for different numbers of markers.

Figure 9 shows the variation trends of the total variances of the translational and rotational measurement errors under different numbers of markers. As the number of markers increases from 4 to 16, both error metrics decrease overall, but the decreasing trend gradually slows down. To further quantify the changes in error, Table 3 and Table 4 list the error metrics for different numbers of markers, the stagewise change in error associated with each addition of two markers, and the baseline-normalized stagewise rate of change calculated using the error metric of the four-marker set as a common reference. According to the definitions provided in the table notes, a negative change indicates a reduction in error, whereas a positive change indicates an increase in error.

As the number of markers increases, the translation estimation error exhibits a clear stepwise decrease. When the number of markers increases from 4 to 6 and from 6 to 8, the translation estimation error decreases by 23.38% and 20.55%, respectively. Increasing the number of markers from 8 to 10 reduces the error by 11.49%. Once the number of markers reaches 12, the error reduction achieved by adding two additional markers falls below 6%. The rotation estimation error exhibits a similar trend. When the number of markers reaches approximately 10–12, the error reduction obtained by adding further markers gradually diminishes. These results indicate that increasing the number of markers can improve pose estimation accuracy, but the resulting improvement gradually decreases as the marker number increases. For the marker configurations considered in the simulations, approximately 12 markers provide a reasonable balance between pose estimation accuracy and marker number.

## 5. Experimental Validation of the Effects of Marker Placement on End-Effector Pose Estimation Error

### 5.1. Experimental Setup and Data Processing Procedure

**Figure 10:**
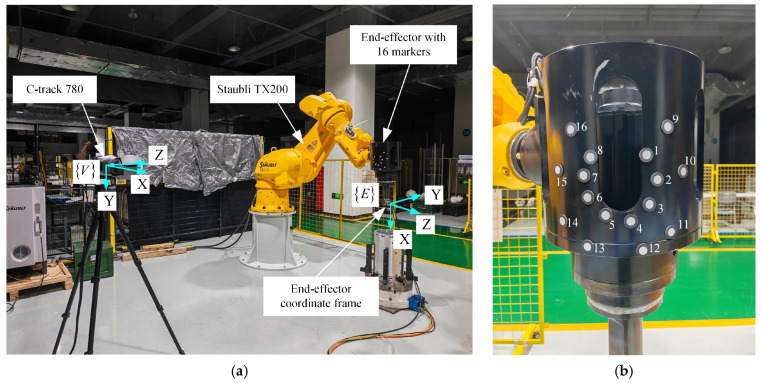
Experimental system and marker arrangement. (**a**) Overview of the experimental system. (**b**) Close-up view and numbering of the 16 markers.

To validate the conclusions obtained from the theoretical analysis and Monte Carlo simulations, a stereo vision measurement system was established to experimentally investigate the effects of marker placement. As shown in Figure 10a, the experimental system mainly consists of an industrial robot, an end-effector, and a stereo camera. The industrial robot is a Stäubli TX200 (Stäubli Faverges SCA, Faverges-Seythenex, France), and the stereo camera is a C-Track 780 manufactured by Creaform (Creaform Inc., Lévis, QC, Canada). Sixteen uncoded circular markers are arranged on the surface of the end-effector facing the stereo camera. The markers are distributed in inner and outer rings. For ease of describing the marker subsets used in the different experiments, the markers are numbered from 1 to 16, as shown in Figure 10b.

The origin of the end-effector coordinate frame $\left\{E\right\}$ is located at the tool center point (TCP), and the three-dimensional coordinates of all markers in $\left\{E\right\}$ are obtained through offline calibration. The stereo camera is fixed in front of the end-effector. The end-effector coordinate frame $\left\{E\right\}$ and the camera coordinate frame $\left\{V\right\}$ are indicated in Figure 10a. The marker-bearing surface is approximately parallel to the image plane of the stereo camera, and all markers are located near the center of the camera field of view. During the experiments, both the stereo camera and the end-effector remain stationary. These experimental conditions minimize the effects of variations in viewing angle, marker occlusion, and image distortion near the image boundaries, thereby highlighting the influence of marker placement on pose estimation errors in rigid-body registration.

Different marker subsets were formed by selecting subsets of the 16 markers described above. For each selected marker subset, the stereo camera continuously measured the three-dimensional coordinates of its markers in the camera coordinate frame, and a total of 1000 measurement sets were collected. For each measurement set, the estimated rotation matrix and translation vector of the end-effector coordinate frame relative to the camera coordinate frame were calculated using the SVD-based rigid registration method described in Appendix A, based on the known marker coordinates in the end-effector coordinate frame and the measured coordinates in the camera coordinate frame. Because the end-effector remained stationary throughout the experiment, the average of the 1000 estimated poses was taken as the experimental reference pose for this stationary configuration. The reference translation was obtained by taking the arithmetic mean of the estimated translation vectors, whereas the reference rotation was calculated using quaternion averaging. Subsequently, the translation and rotation errors of each estimated pose relative to the reference pose were calculated. The translation estimation error metric $E_{t}$ and rotation estimation error metric $E_{r}$ were then determined according to the definitions given in Section 3.

### 5.2. Experimental Analysis of Marker Spatial Distribution Effects

To analyze the effects of marker-set spatial range, spatial distribution balance, and centroid offset distance on end-effector pose estimation errors, one baseline point set and three comparison point sets were constructed. Each point set contained four markers to eliminate the influence of variations in marker number. The marker configurations, geometric properties, and end-effector pose estimation errors of the four point sets are presented in Table 5. The baseline point set consisted of markers 1, 3, 5, and 7. Comparison point set 1 consisted of markers 9, 11, 13, and 15, and had a larger spatial range than the baseline point set. Comparison point set 2 consisted of markers 3, 4, 6, and 11. Its spatial range was similar to that of the baseline point set, but its spatial distribution was substantially less balanced. Comparison point set 3 consisted of markers 1, 3, 5, and 7 and was associated with a virtual end-effector coordinate frame $\left\{E_{virtual}\right\}$. Its centroid offset distance was increased to twice that of the baseline point set, while its spatial range and distribution characteristics remained unchanged.

The spatial range metric of comparison point set 1 was approximately 1.93 times that of the baseline point set, and the minimum eigenvalue of its information matrix was approximately 4.05 times that of the baseline point set. Its translation and rotation estimation errors decreased by 80.88% and 80.19%, respectively. This result indicates that expanding the spatial range of the marker distribution strengthens the geometric constraints imposed by the point set on the end-effector coordinate frame, thereby reducing both translation and rotation estimation errors.

For comparison point set 2, the spatial range metric and centroid offset distance were approximately 94.55% and 91.15% of those of the baseline point set, respectively, indicating that these two geometric properties were similar between the two point sets. However, the minimum eigenvalue of its information matrix was only 16.63% of that of the baseline point set. Compared with the baseline point set, the translation estimation error increased by 28.17%, while the rotation estimation error increased by 48.87%. This result demonstrates that, when the marker number, spatial range, and centroid offset distance are similar, reduced spatial distribution balance substantially weakens the geometric constraint in the least-constrained direction, thereby increasing both translation and rotation estimation errors.

Comparison point set 3 used the same markers as the baseline point set and therefore had the same spatial range and information matrix. When the centroid offset distance was increased to twice that of the baseline point set, the translation estimation error increased by 302.15%, whereas the rotation estimation error remained unchanged. This result indicates that a larger centroid offset distance strengthens the coupling of rotation error into translation error, thereby increasing the translation estimation error without affecting the rotation estimation error itself.

In summary, the experimental results are consistent with the theoretical analysis and Monte Carlo simulation results. Increasing the spatial range of the markers and improving the balance of their spatial distribution are both beneficial for reducing end-effector pose estimation errors. The centroid offset distance primarily affects the translation estimation error and has no apparent effect on the rotation estimation error.

### 5.3. Experimental Analysis of Marker Number Effects

To validate the effect of marker number on end-effector pose estimation errors, seven nested point sets were constructed using the same group of 16 physical markers. The initial point set consisted of markers 1, 3, 5, and 7. Marker pairs (2, 6), (4, 8), (9, 13), (10, 14), (11, 15), and (12, 16) were then added sequentially, increasing the number of markers from 4 to 16. Each pair of newly added markers was approximately centrosymmetric with respect to the point-set centroid to minimize changes in the centroid position as the number of markers increased. In a practical marker layout, increasing the number of markers inevitably changes the spatial range and distribution characteristics of the point set. Therefore, this experiment investigates the combined effect of increasing the number of available markers on pose estimation errors while keeping the point-set centroid approximately unchanged, rather than constructing an ideal single-factor condition in which only the marker number varies. Following the procedure described in Section 5.1, the translation and rotation error metrics were calculated for each nested point set. The definitions of the stagewise error change and rate of change associated with each addition of two markers are consistent with those given in Section 4.4. The experimental results are presented in Table 6 and Table 7, respectively.

When the number of markers increased from 4 to 6, the rotation estimation error decreased by only 4.06%, whereas the translation estimation error increased by 0.25%, which is inconsistent with the theoretical analysis and simulation results. This local fluctuation occurred because the actual stereo vision measurement errors do not strictly satisfy the assumptions of independence, identical distribution, and isotropy. In addition, the experimentally determined reference pose and the finite sample size may introduce small fluctuations, resulting in the observed 0.25% local increase. Because the magnitude of this variation is very small, it does not affect the overall trend of decreasing estimation error as the number of markers increases. When the number of markers increased from 6 to 8 and from 8 to 10, the translation estimation error decreased by 35.25% and 27.44%, respectively, while the rotation estimation error decreased by 22.93% and 26.97%, respectively. These results indicate that increasing the number of markers at this stage substantially improved pose estimation accuracy. When the marker number increased from 10 to 12, from 12 to 14, and from 14 to 16, the translation estimation error decreased by 8.81%, 14.23%, and 3.96%, respectively, while the rotation estimation error decreased by 17.65%, 13.14%, and 3.00%, respectively. Compared with the preceding stage, the improvement in pose estimation accuracy obtained by adding further markers became less pronounced.

Considering the changes in both translation and rotation estimation errors, under the marker layout and measurement conditions used in this experiment, approximately 12 markers provide a reasonable trade-off between pose estimation accuracy and marker number. Although adding further markers can still reduce the errors, the resulting improvement in pose estimation accuracy is substantially diminished.

## 6. Conclusions

This paper investigates the effects of marker placement factors on end-effector pose estimation error, with the aim of improving end-effector pose estimation accuracy in industrial robot visual servoing. The main conclusions are as follows:

(1) A propagation relationship from marker measurement errors to end-effector pose estimation errors was established. The covariance expressions of the translational and rotational errors were derived.
(2) For rotational estimation error, a more balanced marker spatial distribution and a larger marker-set spatial range are more favorable for reducing the rotational estimation error. The centroid offset does not affect the rotational estimation error.
(3) For translational estimation error, a larger marker-set spatial range and a smaller centroid offset distance are more favorable for reducing the translational estimation error. The effect of marker spatial distribution balance on the translational estimation error does not have general monotonicity. It depends on the relative relationship between the centroid offset vector and the characteristic directions of the marker set. The closer the principal axis direction of the marker set is to the centroid offset direction, the more favorable it is for reducing the translational error.
(4) Adding markers to an existing point set is generally beneficial for reducing the end-effector pose estimation error. The simulation and experimental results show that, as the number of markers increases from 4 to 16, both translational and rotational estimation errors exhibit an overall decreasing trend, but the decreasing amplitude gradually becomes smaller. Under the end-effector scale and marker placement conditions considered in this paper, 12 markers can be regarded as a reasonable placement number. Further increasing the number of markers yields only limited additional improvement.

The marker-placement analysis in this study is conducted under the assumption that the measurement errors of individual markers are independent, identically distributed, and isotropic. In practical stereo vision measurement, marker errors may vary with spatial position, observation distance, viewing angle, image quality, and occlusion, and may exhibit heterogeneous, anisotropic, correlated, or biased characteristics. Under such conditions, end-effector pose uncertainty is jointly determined by the spatial arrangement of the markers and the statistical characteristics of their measurement errors. Future work will investigate error propagation models under more general marker-error characteristics and analyze the effects of marker placement and marker number on end-effector pose estimation errors.

## Figures and Tables

**Table 1:** Comparison of pose estimation errors between simulation and the analytical model.

	Simulation	Analytical	Relative Difference ^(1)^
$\mathrm{Var}\left(\delta t_{x}\right)/\left(\times 10^{-3}\;{\mathrm{mm}}^{2}\right)$	2.79	2.71	2.90%
$\mathrm{Var}\left(\delta t_{y}\right)/\left(\times 10^{-3}\;{\mathrm{mm}}^{2}\right)$	3.48	3.41	2.15%
$\mathrm{Var}\left(\delta t_{z}\right)/\left(\times 10^{-3}\;{\mathrm{mm}}^{2}\right)$	1.44	1.46	1.22%
$\mathrm{Var}\left(\delta \theta_{x}\right)/\left(\times 10^{-8}\;{\mathrm{rad}}^{2}\right)$	9.19	9.25	0.68%
$\mathrm{Var}\left(\delta \theta_{y}\right)/\left(\times 10^{-8}\;{\mathrm{rad}}^{2}\right)$	5.16	5.08	1.59%
$\mathrm{Var}\left(\delta \theta_{z}\right)/\left(\times 10^{-8}\;{\mathrm{rad}}^{2}\right)$	9.43	9.51	0.93%
Relative Frobenius norm error ^(1)^	2.79%		

(1): Relative differences and the relative Frobenius norm error were calculated using unrounded values.

**Table 2:** Marker coordinates used for analyzing the effect of the number of markers.

i	$p_{i}$/(mm)	i	$p_{i}$/(mm)
1	(123, 109, −80)	9	(−70, −40, −50)
2	(110, −111, −322)	10	(66, 43, −345)
3	(−109, 118, −311)	11	(135, 95, −220)
4	(−124, −116, −87)	12	(−130, −98, −176)
5	(160, −50, −180)	13	(−120, 145, −235)
6	(−154, 54, −217)	14	(117, −154, −182)
7	(40, 155, −245)	15	(25, −165, −240)
8	(−44, −150, −150)	16	(−20, 160, −165)

**Table 3:** Simulation results of translational estimation error for different numbers of markers.

Number of Markers n	$E_{t}/(\times 10^{-3}\;{\mathrm{mm}}^{2})$	$D_{t}/(\times 10^{-3}\;{\mathrm{mm}}^{2})$ ^(1)^	$\eta_{E_{t}}$ ^(1)^
4	14.93	-	-
6	11.44	−3.49	−23.38%
8	8.37	−3.07	−20.55%
10	6.66	−1.72	−11.49%
12	5.87	−0.79	−5.26%
14	5.04	−0.83	−5.59%
16	4.53	−0.51	−3.40%

(1): $D_{t}^{\left(n\right)}=E_{t}^{\left(n\right)}-E_{t}^{\left(n-2\right)}$, and $\eta_{E_{t}}^{\left(n\right)}=\left(D_{t}^{\left(n\right)}/E_{t}^{\left(4\right)}\right)\times 100\%$. The values of $D_{t}$ and $\eta_{E_{t}}$ were calculated using unrounded values.

**Table 4:** Simulation results of rotational estimation error for different numbers of markers.

Number of Markers n	$E_{r}/(\times 10^{-7}\;{\mathrm{rad}}^{2})$	$D_{r}/(\times 10^{-7}\;{\mathrm{rad}}^{2})$ ^(1)^	$\eta_{E_{r}}$ ^(1)^
4	2.82	-	-
6	2.22	−0.60	−21.32%
8	1.66	−0.56	−19.79%
10	1.38	−0.28	−9.98%
12	1.22	−0.16	−5.69%
14	1.01	−0.21	−7.39%
16	0.89	−0.12	−4.18%

(1): $D_{r}^{\left(n\right)}=E_{r}^{\left(n\right)}-E_{r}^{\left(n-2\right)}$, and $\eta_{E_{r}}^{\left(n\right)}=\left(D_{r}^{\left(n\right)}/E_{r}^{\left(4\right)}\right)\times 100\%$. The values of $D_{r}$ and $\eta_{E_{r}}$ were calculated using unrounded values.

**Table 5:** Point-set configurations, geometric properties, and pose estimation errors in the experiment on the effects of marker spatial distribution.

	Baseline Point Set	Comparison Point Set 1	Comparison Point Set 2	Comparison Point Set 3
Marker IDs	(1, 3, 5, 7)	(9, 11, 13, 15)	(3, 4, 6, 11)	(1, 3, 5, 7)
Associated coordinate frame	$\left\{E\right\}$	$\left\{E\right\}$	$\left\{E\right\}$	$\left\{E_{virtual}\right\}$
$\lambda_{\min}$	4169.24	16,880.67	693.16	4169.24
$S_{p}/\left(\mathrm{mm}\right)$	101.93	196.41	96.37	101.93
$\left\|c\right\|/\left(\mathrm{mm}\right)$	462.48	449.28	421.53	924.96
$E_{t}/(\times 10^{-3}\;{\mathrm{mm}}^{2})$	20.52	3.93	26.31	82.54
$\rho_{E_{t}}$ ^(1)^	-	−80.88%	28.17%	302.15%
$E_{r}/(\times 10^{-7}\;{\mathrm{rad}}^{2})$	1.47	0.29	2.19	1.47
$\rho_{Er}$ ^(1)^	-	−80.19%	48.87%	0.00%

(1): $\rho_{E_{t}}$ and $\rho_{Er}$ denote the relative changes in the translational and rotational error metrics, respectively, with respect to the baseline marker set. Positive values indicate increases in error, whereas negative values indicate decreases. Both $\rho_{E_{t}}$ and $\rho_{Er}$ were calculated using unrounded values.

**Table 6:** Experimental results of translational estimation error for different numbers of markers.

Number of Markers n	$E_{t}/(\times 10^{-3}\;{\mathrm{mm}}^{2})$	$D_{t}/(\times 10^{-3}\;{\mathrm{mm}}^{2})$ ^(1)^	$\eta_{E_{t}}$ ^(1)^
4	20.52	-	-
6	20.58	0.05	0.25%
8	13.34	−7.24	−35.25%
10	7.71	−5.63	−27.44%
12	5.90	−1.81	−8.81%
14	2.98	−2.92	−14.23%
16	2.17	−0.81	−3.96%

(1): $D_{t}^{\left(n\right)}=E_{t}^{\left(n\right)}-E_{t}^{\left(n-2\right)}$, and $\eta_{E_{t}}^{\left(n\right)}=\left(D_{t}^{\left(n\right)}/E_{t}^{\left(4\right)}\right)\times 100\%$. The values of $D_{t}$ and $\eta_{E_{t}}$ were calculated using unrounded values.

**Table 7:** Experimental results of rotational estimation error for different numbers of markers.

Number of Markers n	$E_{r}/(\times 10^{-8}\;{\mathrm{rad}}^{2})$	$D_{r}/(\times 10^{-8}\;{\mathrm{rad}}^{2})$ ^(1)^	$\eta_{E_{r}}$ ^(1)^
4	14.68	-	-
6	14.08	−0.60	−4.06%
8	10.72	−3.37	−22.93%
10	6.76	−3.96	−26.97%
12	4.17	−2.59	−17.65%
14	2.24	−1.93	−13.14%
16	1.80	−0.44	−3.00%

(1): $D_{r}^{\left(n\right)}=E_{r}^{\left(n\right)}-E_{r}^{\left(n-2\right)}$, and $\eta_{E_{r}}^{\left(n\right)}=\left(D_{r}^{\left(n\right)}/E_{r}^{\left(4\right)}\right)\times 100\%$. The values of $D_{r}$ and $\eta_{E_{r}}$ were calculated using unrounded values.

## Data Availability

Data will be made available on request.

## Appendix A. SVD-Based Rigid Registration Method

Given n corresponding markers, let $p_{i}$ denote the known coordinate of the i-th marker in the end-effector coordinate system and let $q_{i}$ denote its measured coordinate in the camera coordinate system. The rigid registration problem is formulated as:

(A1) $$\underset{\hat{R},\hat{t}}{\min}\sum_{i=1}^{n}{\left\|q_{i}-\left(\hat{R}p_{i}+\hat{t}\right)\right\|}^{2},\hat{R}\in SO\left(3\right)$$

First, the centroids of the two point sets are calculated as:

(A2) $$\bar{p}=\frac{1}{n}\sum_{i=1}^{n}p_{i},\bar{q}=\frac{1}{n}\sum_{i=1}^{n}q_{i}$$

The centered coordinates are then obtained as:

(A3) $${\tilde{p}}_{i}=p_{i}-\bar{p},{\tilde{q}}_{i}=q_{i}-\bar{q}$$

The cross-covariance matrix is constructed as:

(A4) $$H=\sum_{i=1}^{n}{\tilde{q}}_{i}{\tilde{p}}_{i}^{T}$$

Applying singular value decomposition gives:

(A5) $$H=U\Sigma V^{T}$$

To ensure that the estimated rotation matrix represents a proper rotation without reflection, define:

(A6) $$D=\mathrm{diag}\left(1,1,\det\left(UV^{T}\right)\right)$$

The estimated rotation matrix and translation vector are then calculated as:

(A7) $$\hat{R}=\mathrm{UD}V^{T},\hat{t}=\bar{q}-\hat{R}\bar{p}$$
